# Reducing Noise and Alarm Burden in Adult Intensive Care Units: An Integrative Review of Interventions and Implications for Nursing Management

**DOI:** 10.1155/jonm/9617407

**Published:** 2026-10-29

**Authors:** Wei Jun Dan Ong, Michael Camba Vidanes, Woon Hean Keenan Chong, Faheem Ahmed Khan, Patricia Leong, Lawrence Ace Matunan Azul, Adrian Ujin Yap

**Affiliations:** ^1^ Respiratory Therapy Department Ng Teng Fong General Hospital National University Health System Singapore nuhs.edu.sg; ^2^ Department of Health Sciences College of Health Sciences Rush University Chicago Illinois USA rush.edu; ^3^ Department of Intensive Care Medicine Ng Teng Fong General Hospital National University Health System Singapore nuhs.edu.sg; ^4^ Nursing Administration Ng Teng Fong General Hospital National University Health System Singapore nuhs.edu.sg; ^5^ Clinical Research Unit Ng Teng Fong General Hospital National University Health System Singapore nuhs.edu.sg; ^6^ National Dental Research Institute Singapore, National Dental Centre Singapore, and Duke-NUS Medical School Singapore Health Services Singapore singhealth.com.sg

**Keywords:** clinical alarms, intensive care units, noise, nursing management, patient safety

## Abstract

**Aim:**

To synthesise evidence on noise‐reduction and alarm‐management strategies in adult intensive care units and examine their implications for nursing management.

**Background:**

ICU noise and alarm burden present challenges for patient rest, clinical surveillance, nursing workflow and staff experience. Intervention effectiveness and sustainability remain uncertain.

**Evaluation:**

An integrative review guided by Whittemore and Knafl’s framework synthesised empirical, implementation and review‐level evidence identified through PubMed, Scopus, CINAHL and reference screening.

**Key Issues:**

Interventions addressed alarm management, environmental modification, quiet‐hour practices, staff engagement and multicomponent implementation. Reported benefits varied across studies, and available evidence did not establish the superiority of bundled approaches. Direct evidence concerning workforce well‐being, nursing leadership, clinical surveillance and sustained improvement was limited.

**Conclusion:**

Quiet ICU interventions may improve selected acoustic and alarm‐related outcomes, but their comparative effectiveness and long‐term sustainability remain uncertain.

**Implications for Nursing Management:**

Nurse leaders may consider context‐sensitive interventions supported by alarm governance, workflow integration, staff participation and ongoing evaluation. Future studies should assess nursing workload, clinical surveillance, patient safety and sustainability alongside acoustic outcomes.

## 1. Introduction

Intensive care units (ICUs) are acoustically complex clinical environments in which patients and staff are continuously exposed to sounds from alarms, equipment, conversations and routine care activities [1, 2]. Although ICU noise is often framed as an environmental problem, it is also a nursing management issue because it affects patient safety, staff concentration, alarm recognition, communication, workflow coordination and workforce well‐being [3–5]. From a management perspective, noise and alarm burden represent recurrent workplace exposures shaped by staffing patterns, alarm governance, environmental design, bedside behaviours, leadership priorities and the extent to which safe work systems are embedded into routine practice. In adult ICUs, sound levels frequently exceed recommended thresholds, particularly during night‐time periods when uninterrupted rest and recovery are most needed [2, 6].

Excessive ICU noise has implications for both patients and staff. For patients, noise may disrupt sleep, increase physiological stress and contribute to adverse outcomes during recovery [2, 7]. For staff, especially nurses working in alarm‐dense environments, excessive sound may impair concentration, increase cognitive strain, disrupt communication and complicate the interpretation of clinically important auditory cues [5, 8, 9]. These effects are particularly relevant in high‐acuity settings where alarm recognition, bedside monitoring, communication, task prioritisation and rapid decision‐making occur simultaneously under time pressure [3, 10, 11]. ICU noise and alarm burden should therefore be understood not only as patient‐safety concerns but also as workforce, governance and leadership challenges requiring structured managerial action.

The concept of a Quiet ICU emerged from broader work on healing environments, evidence‐based design and human factors, which emphasise the relationship between environmental stressors and health outcomes [1]. Early approaches focused primarily on reducing ambient noise, improving the physical design and creating calmer recovery environments. However, contemporary ICU noise is not generated solely by the built environment. It arises from the interaction of alarm systems, bedside workflow, communication patterns, staff behaviours, patient acuity, equipment use and organisational culture [3, 4, 12, 13]. Sustainable noise reduction, therefore, requires more than physical modification or individual awareness; it requires leadership, governance, feedback and integration into routine nursing and unit‐management processes.

Many ICUs have introduced noise‐reduction initiatives, often described as Quiet ICU programmes [12, 13]. These interventions commonly include alarm optimisation, staff education, behavioural reinforcement, quiet‐hour practices, environmental modifications and multicomponent implementation bundles [3, 10, 12]. Such strategies aim to reduce sound exposure while supporting patient rest, staff functioning, alarm relevance and safer care delivery [1, 11]. However, reported effectiveness is variable, and long‐term sustainability remains difficult in high‐acuity clinical environments [5, 13]. From a nursing management perspective, this variability is important because interventions that are not aligned with staffing realities, bedside workflow, escalation processes and leadership support may be difficult to sustain beyond the initial implementation period.

A nursing leadership–focused synthesis is warranted because nurses are central to both the ICU soundscape and the implementation of noise‐reduction strategies. Nurses work continuously within alarm‐dense environments, respond to bedside alarms, coordinate care activities that generate procedural noise and manage competing demands while maintaining patient surveillance [5, 8, 10]. Unlike clinicians who enter and leave the ICU intermittently, nurses experience sustained exposure to the acoustic environment and are often responsible for operationalising quiet‐hour practices, alarm review, patient rest promotion and escalation decisions at the bedside [4, 11]. Nurse managers and unit leaders therefore have a key role in ensuring that Quiet ICU strategies are feasible, acceptable, monitored and sustained. This requires moving beyond awareness campaigns towards embedded governance mechanisms, including alarm‐management protocols, staff engagement, audit and feedback, workflow redesign and leadership reinforcement.

Previous reviews have largely focused on sound levels, alarm fatigue, patient sleep or environmental interventions [6, 9]. However, the implications of these findings for nursing management remain less clearly established, particularly the relationships among intervention design, bedside workflow, staff experience, alarm governance and implementation sustainability. Improvements in acoustic or alarm‐related outcomes should not be assumed to translate directly into reduced nursing workload, better workforce well‐being or enhanced clinical surveillance. An integrative review permits the analysis of quantitative, qualitative, implementation and conceptual sources while distinguishing directly reported outcomes from broader management interpretations.

Accordingly, this integrative review synthesises recent evidence on interventions used to reduce environmental noise and alarm burden in adult ICUs. The review examines the main intervention strategies; reported patient‐, staff‐, and system‐level outcomes; and implications for nursing management, workforce well‐being, workflow design and implementation sustainability. The review asked: what interventions have been used to reduce environmental noise and alarm burden in adult ICUs? what outcomes have been reported? and what implications do these findings have for nursing leadership and management practice?

## 2. Methods

### 2.1. Design

An integrative review design was adopted because the ICU noise and alarm‐management literature includes quantitative, qualitative, mixed‐methods, observational, quality‐improvement and implementation research alongside existing evidence syntheses. The purpose was not to estimate one pooled intervention effect but to examine intervention content, observed outcomes, feasibility and potential implications for nursing management. Primary empirical studies were distinguished from review‐level sources when interpreting findings to avoid treating different levels of evidence as interchangeable.

The review was guided by Whittemore and Knafl’s five‐stage methodology: problem identification, literature search, data evaluation, data analysis and presentation [14]. Relevant PRISMA 2020 principles were applied to report study identification, screening, eligibility assessment and selection [15]. The research project was initially registered with PROSPERO on 23 October 2025 (CRD420251172154) as a systematic review. The completed review adopted an integrative approach to accommodate heterogeneous evidence, including quantitative, qualitative, observational, implementation and review‐level literature. Accordingly, the eligibility criteria, search scope and methodological appraisal approach differed from those specified in the original registered protocol.

### 2.2. Search Strategy

A structured literature search was conducted across PubMed, Scopus and CINAHL to identify peer‐reviewed studies examining ICU noise, alarm burden and intervention strategies intended to reduce environmental sound exposure or improve alarm management. These databases were selected to provide focused coverage of biomedical, nursing, allied health and interdisciplinary health sciences literature relevant to adult intensive care practice and nursing management. Backward citation screening of included articles was used to identify relevant studies not captured through database indexing.

Search terms were developed around four core concepts: intensive care, noise or sound exposure, alarm burden or alarm fatigue and intervention or management strategy. A representative Boolean search string was as follows:

(‘intensive care unit’ OR ‘critical care’) AND (‘noise’ OR ‘sound level’ OR ‘acoustic environment’ OR ‘alarm fatigue’) AND (‘intervention’ OR ‘alarm management’ OR ‘noise reduction’ OR ‘quiet hour’ OR ‘sound masking’).

Searches were limited to English‐language, peer‐reviewed studies published from January 2019 to August 2025. This period was selected to capture contemporary ICU environments, recent alarm‐management practices, current implementation approaches and post‐pandemic workforce and workflow conditions relevant to nursing management. The final database search was conducted on 31 January 2026 to identify records from this eligibility period that may have been indexed after the initial search. Full database‐specific search strategies are presented in Table S1. Retrieved records were imported into Covidence for deduplication, title and abstract screening and full‐text assessment using a structured screening workflow.

The search focused on ICU noise, acoustic exposure, alarm burden and related intervention or management strategies. Nursing leadership, workforce well‐being and governance and sustainability were examined when reported by studies identified through this search but were not comprehensively searched as independent domains. Therefore, this review should not be interpreted as a systematic account of all ICU nursing management or occupational well‐being research.

### 2.3. Study Selection

Study selection followed a structured two‐stage screening process informed by PRISMA principles and Whittemore and Knafl’s integrative review methodology [14, 15]. A three‐reviewer protocol was used throughout the review. Two reviewers independently conducted title and abstract screening, full‐text eligibility assessment and methodological appraisal. A third reviewer acted as arbitrator and resolved discrepancies through discussion and consensus.

In Stage 1, the two primary reviewers independently screened titles and abstracts against predefined eligibility criteria. In Stage 2, articles judged potentially relevant underwent independent full‐text assessment by the same two reviewers. Reasons for exclusion at the full‐text stage were documented prospectively using predefined categories [15]. Disagreements at either stage were resolved through discussion and, where necessary, referral to the third reviewer.

Publications were considered eligible if they examined ICU acoustic environments, noise exposure, alarm burden, intervention or implementation strategies, or related patient‐, staff‐, and organisational outcomes relevant to adult critical care. The integrative review considered quantitative, qualitative, observational, mixed‐methods, participatory design, implementation and review‐level literature. Primary studies were distinguished according to whether they evaluated an intervention, characterised environmental exposure, investigated associations with clinical outcomes or explored staff experiences and implementation processes. Observational and acoustic characterisation studies were treated as contextual evidence rather than evaluations of intervention effectiveness. Review‐level publications were included to provide conceptual and implementation context rather than independent evidence of intervention effectiveness. Observational and descriptive publications were eligible when they contributed directly relevant information concerning ICU acoustic exposure, noise sources, alarm burden, associated clinical or workforce outcomes or factors influencing noise‐management practices. These publications were distinguished from intervention evaluations during data extraction and synthesis. Publications were excluded when they lacked sufficient relevance to ICU noise or alarm burden and their associated clinical, environmental or management implications, or when they were conducted exclusively outside the eligible adult critical care setting. Evidence types were interpreted according to their methodological characteristics to avoid attributing intervention effects to observational or contextual findings.

To minimise the risk of double‐counting, primary empirical publications and review‐level sources were distinguished throughout the integrative synthesis. Comparison of the five review‐level publications with the eight primary reports identified one confirmed direct overlap: Souza et al. [13] was included in the systematic review by Vreman et al. [6]. In addition, Armbruster et al. [16] and Witek et al. [17] reported complementary staff‐related and acoustic outcomes from the same registered ICU noise‐management programme (DRKS00025835). These publications were retained because they contributed distinct outcome information but were not interpreted as independent evaluations of separate intervention programmes. Primary empirical publications formed the principal basis for interpreting directly observed outcomes, whereas review‐level sources contributed contextual and conceptual evidence. Findings originating from overlapping studies or research programmes were not treated as independent confirmation of intervention effectiveness.

The overlap assessment was based on published included‐study lists and available information concerning the underlying evidence. For review‐level publications without clearly reproducible included‐study inventories, including Pal et al. [4], complete overlap ascertainment was not possible. These publications were therefore used for general contextual interpretation rather than as independent evidence of intervention effects. The synthesis also distinguished observational and acoustic characterisation studies from intervention evaluations. In addition, the inclusion of neonatal ICU settings within the research programme reported by Armbruster et al. [16] and Witek et al. [17] was considered when interpreting applicability to adult critical care. The study‐selection process was reported using a PRISMA‐informed flow diagram (Figure 1) [15], and the published‐source overlap assessment is documented in Table S2.

**FIGURE 1:**
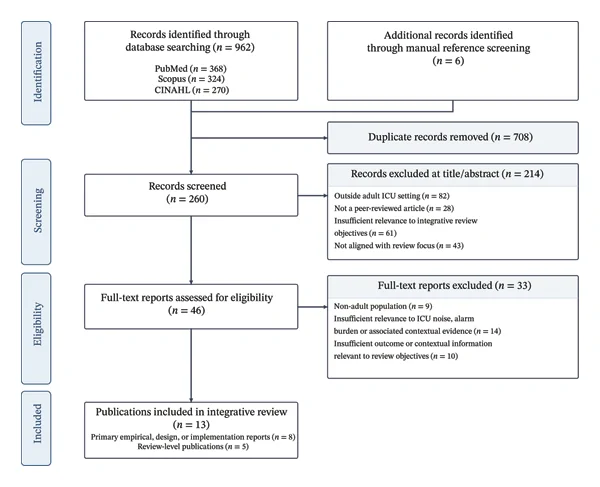
PRISMA‐informed flow diagram of the study selection process. This figure presents the systematic process of study identification, screening, eligibility assessment and inclusion for the integrative review. A total of 962 records were identified through database searching (CINAHL, PubMed, and Scopus), with six additional records identified through manual reference screening. Following duplicate removal (*n* = 708), 260 records were screened by title and abstract, resulting in 214 exclusions. Forty‐six full‐text articles were assessed for eligibility, of which 33 were excluded based on predefined inclusion and exclusion criteria. Thirteen publications were included in the final integrative synthesis, comprising eight primary empirical, design or implementation reports and five review‐level publications.

### 2.4. Data Evaluation

Methodological quality was evaluated using design‐appropriate Joanna Briggs Institute (JBI) critical appraisal tools and the Mixed Methods Appraisal Tool (MMAT) for eligible mixed‐methods studies [18, 19]. Each publication was assessed using an appraisal approach appropriate to its study design and evidentiary contribution, with consideration of methodological transparency, sampling, outcome measurement, potential confounding, analytical rigour and the appropriateness of conclusions. Two reviewers independently conducted methodological appraisal, with disagreements resolved through discussion or consultation with a third reviewer. For publications not directly compatible with established JBI or MMAT instruments, methodological limitations were evaluated using a structured, design‐informed narrative approach. Appraisal findings informed the interpretation of study credibility, applicability and methodological limitations without generating categorical certainty‐of‐evidence ratings or serving as automatic exclusion criteria. Study‐level appraisal findings and the instruments or approaches applied are presented in Table S3.

### 2.5. Data Extraction

Data were extracted into a structured matrix developed from the review framework. Extracted domains included author, year, country, ICU setting, study design, sample or population, intervention or strategy, comparator where applicable, outcome measures, patient‐level outcomes, staff‐level outcomes, system‐level outcomes, implementation processes, barriers and facilitators, appraisal findings and key implications for nursing management.

Data extraction was conducted by one reviewer and checked by a second reviewer for completeness and accuracy. Any uncertainties were resolved through discussion within the review team. This process was used to ensure consistency across heterogeneous study types and to support transparent comparison of intervention characteristics, outcomes and implementation relevance.

### 2.6. Data Analysis and Synthesis

Because the included literature was heterogeneous in study design, intervention intensity, comparator use, outcome measurement and reporting, statistical pooling was not appropriate. Findings were therefore synthesised using thematic narrative synthesis, consistent with integrative review methodology [14].

Primary empirical studies were given greater interpretive weight when drawing conclusions about intervention effects and practice implications. Review‐level articles were used primarily to contextualise recurring patterns in the field and to identify broader implementation considerations. During synthesis, particular attention was paid to findings relevant to nursing management, including workforce burden, workflow feasibility, leadership support, alarm governance, implementation barriers, staff engagement and sustainability.

The synthesis proceeded in four steps. First, the extracted data were reviewed repeatedly to become familiar with the included studies. Second, studies were compared according to intervention focus, mechanism of action, reported outcomes, staff and patient implications and implementation barriers. Third, recurring patterns were grouped into preliminary thematic categories. Fourth, these categories were iteratively refined through team discussion into the final intervention framework [14].

The thematic synthesis differentiated three principal intervention domains and two cross‐cutting implementation dimensions. The intervention domains comprised alarm‐management strategies, environmental modifications and quiet‐hour or workflow interventions. These domains represented the principal targets of noise‐ and alarm‐reduction activities. Staff engagement and culture change were interpreted as implementation processes, whereas multicomponent bundled approaches represented configurations combining two or more intervention strategies. The categories were not mutually exclusive because individual publications frequently addressed multiple environmental, technological, behavioural and organisational components. This classification enabled intervention content, directly reported outcomes and nursing management implications to be examined separately.

### 2.7. Statistical Analysis and Sample‐Size Considerations

No meta‐analysis, pooled effect estimation or review‐level hypothesis testing was undertaken because the included publications differed substantially in study design, intervention content, comparator availability, outcome measurement and reporting. Findings were integrated using descriptive comparison and thematic narrative synthesis. Accordingly, no review‐level statistical significance threshold or power calculation was applicable. The number of included publications was determined by the eligibility criteria and literature search rather than an a priori sample‐size calculation. Where available, quantitative results reported by individual studies were interpreted in relation to their original designs and methodological limitations.

### 2.8. Ethical Considerations

This integrative review synthesised evidence from previously published literature and did not involve primary participant recruitment, collection of identifiable patient information or delivery of a clinical intervention. Institutional ethics approval and informed consent were therefore not required. An ethics approval number was not applicable.

## 3. Results

### 3.1. Search Results and Characteristics of Included Studies

A total of 962 records were identified through database searching: PubMed (*n* = 368), Scopus (*n* = 324) and CINAHL (*n* = 270). An additional six records were identified through manual reference screening. After removing 708 duplicate records, 260 records remained for title and abstract screening. Of these, 214 records were excluded. Forty‐six full‐text publications were assessed for eligibility, of which 33 were excluded: non‐adult or otherwise ineligible populations (*n* = 9); insufficient relevance to ICU noise or alarm burden and associated intervention, implementation or contextual evidence (*n* = 14); and insufficient outcome or contextual information relevant to the review objectives (*n* = 10). Thirteen publications were included in the final integrative synthesis, comprising eight primary empirical or implementation reports and five review‐level sources (Figure 1).

The 13 included publications represented a heterogeneous evidence base addressing ICU noise reduction, alarm burden, staff experience, environmental design and implementation strategies in adult critical care settings. Eight publications were primary empirical, design or implementation reports, encompassing observational, qualitative, mixed‐methods; participatory design; best‐practice implementation; and pre‐post evaluations. Five were review‐level publications, including integrative, narrative, systematic, comprehensive literature review and meta‐synthesis approaches. Primary empirical publications and review‐level sources were distinguished throughout the synthesis. In addition, two primary publications reported complementary outcomes from the same registered ICU noise‐management programme and were not treated as independent intervention evaluations.

Study populations and samples varied by design and included ICU nurses, multidisciplinary healthcare professionals, ICU patients, patient stays, ICU bed spaces and environmental sound measurements. Review‐level sources synthesised evidence on ICU alarm management, noise‐mitigating interventions, built‐environment design, alarm fatigue and qualitative nursing experiences. Reported outcomes included sound‐level measures, false alarms, alarm duration, nurse response time, alarm fatigue, subjective noise exposure, noise‐related strain, sleep‐related outcomes, delirium‐related outcomes, environmental comfort, workflow feasibility, adherence to best‐practice criteria and implementation follow‐up.

Five thematic categories were identified: alarm‐management strategies, environmental modifications, quiet‐hour and workflow interventions, staff engagement and culture change and multicomponent bundled approaches. The first three categories represented intervention domains, whereas the remaining two reflected cross‐cutting implementation processes and intervention configurations. Because individual publications frequently addressed multiple categories, these were interpreted as overlapping rather than mutually exclusive. Table 1 summarises the characteristics, evidence roles and methodological limitations of the included publications. Table 2 presents the thematic synthesis, distinguishing directly reported findings from implementation considerations and nursing management implications.

**TABLE 1:** Characteristics and key findings of publications included in the quiet ICU integrative review.

Publication study	Study design and setting	Sample	Intervention or research focus	Directly reported findings	Methodological limitations
Armbruster et al. [16]	Prospective longitudinal observational study; Germany; three ICUs, including adult and neonatal settings	179 healthcare professionals	Staff noise exposure and unit‐based noise‐management programme	High perceived noise exposure; improvement in some perceptions of the occupational sound environment, without significant improvement in overall subjective noise exposure	Single‐centre design; possible temporal confounding; shares a research programme with Witek et al.
Li et al. [3]	Integrative review; China	Seven included studies	Intelligent ICU alarm‐management interventions	Review‐level findings described reductions in false alarms, alarm duration, response time and nurse alarm fatigue	Heterogeneous interventions and outcomes; secondary evidence rather than an independent intervention evaluation
Lu et al. [10]	Qualitative interview study; Taiwan; medical and surgical ICU	15 ICU nurses	Nurses’ experiences and coping strategies in alarm management	Identified strategies involving alarm recognition, team monitoring, sensory discrimination and reflective learning	Small, context‐specific qualitative sample; no intervention‐effect estimation
Pal et al. [4]	Narrative review; India	Not applicable	ICU noise sources and consequences for patients and caregivers	Described ICU noise as a persistent environmental concern with potential physical and psychological consequences	Limited transparency of search and study selection; no independently estimated effects
Sangari et al. [7]	Prospective observational cohort study; United States; surgical and trauma ICU	143 patient stays; more than 111 million sound and light measurements	Association between ICU sound/light exposure and delirium	Sound and light exposure were associated with incidence of delirium	Observational associations; potential confounding; no acoustic intervention evaluated
Souza et al. [13]	Best‐practice implementation project; Brazil; adult ICU	Seven audited best‐practice criteria	Quiet‐hour practices and noise‐reduction implementation	Improved adherence to several noise‐reduction practices; achieving recommended sound thresholds remained challenging	Local implementation without a strong independent comparator; also included in Vreman et al.
Tahvili et al. [2]	Prospective acoustic cohort study; United Kingdom; six‐bed ICU bay	11 patients; 16 days of monitoring	Acoustic exposure and noise‐source characterisation	Sound levels exceeded recommended guidance; common sources included patient care, repositioning, medication administration and suctioning	Single setting and short observation period; no intervention tested
Tronstad et al. [12]	Multistage mixed‐methods participatory design study; Australia	Two redesigned ICU bed spaces	Patient‐centred ICU environmental design	Described participatory design processes and environmental features intended to support patient recovery	Design‐focused evidence; clinical effects of individual environmental changes were not established
Uçak et al. [8]	Convergent parallel mixed‐methods study; Türkiye; adult ICU	67 nurses in quantitative phase; 14 in qualitative phase	Alarm fatigue and nurses’ strategies to manage alarm burden	Alarm fatigue was identified among nurses, alongside practical behavioural and organisational coping strategies	Site‐specific sample; self‐reported and qualitative findings; no causal intervention comparison
Verderber et al. [1]	Comprehensive built‐environment literature review; international evidence	135 literature sources	ICU architecture and recovery‐supportive environmental design	Identified acoustic conditions among design considerations relevant to healing‐oriented ICU environments	Broad, heterogeneous evidence; no isolated noise‐intervention effect estimate
Vreman et al. [6]	Systematic review; Netherlands	25 included studies	ICU noise‐reduction interventions	Selected interventions reduced mean sound levels, but findings were heterogeneous and sustainability remained uncertain	Variation in intervention design and outcomes; includes Souza et al. [13]
Witek et al. [17]	Non‐randomised before–after implementation study; Germany; three ICUs, including a neonatal ICU	Three ICU units; 12‐ and 24‐week assessments	Multicomponent ICU noise‐management programme	Modest reductions in sound levels after implementation; benefits were less durable at follow‐up and smaller after workload adjustment	No independent randomised comparator; mixed adult/neonatal settings; same programme as Armbruster et al.
Xu et al. [11]	Qualitative systematic review and meta‐synthesis; China	11 included qualitative studies	ICU nurses’ alarm‐management experiences and strategies	Synthesised findings concerning alarm fatigue, alarm reliability, training, working environment and staff support	Secondary qualitative evidence; heterogeneity and no direct intervention‐effect estimation

*Note:* LAeq = equivalent continuous sound level, where applicable. Primary empirical and implementation reports were distinguished from review‐level publications. Review‐level findings were used for contextual synthesis rather than as independent confirmation of primary intervention effects. Armbruster et al. and Witek et al. reported complementary findings from the same registered programme, while Souza et al. was also included in Vreman et al. Detailed methodological appraisal findings are presented in Table S3.

Abbreviation: ICU = intensive care unit.

**TABLE 2:** Thematic synthesis of quiet ICU intervention strategies, mechanisms, outcomes and implementation considerations.

Intervention category	Representative strategies	Proposed mechanism or rationale	Reported outcomes	Implementation considerations
Alarm management strategies	Individualised alarm thresholds; alarm parameter review; escalation protocols; remote alarm monitoring; filtering of non‐actionable alarms	May reduce non‐actionable alarms and improve the prioritisation of clinically important alarm notifications	Review‐level evidence described potential reductions in non‐actionable alarms and alarm duration. Primary studies documented nurses’ experiences and alarm‐management strategies; safety effects remain uncertain	Requires standardised protocols, staff training, device interoperability and sustained governance; effectiveness is limited if poorly integrated into workflow
Environmental modifications	Sound‐absorbing materials; acoustic panels; silent flooring; door management; signage; ICU design optimisation	May reduce sound transmission, reverberation and environmental acoustic exposure	Observational and design studies characterised acoustic exposure, noise sources and environmental design considerations. Direct effectiveness evidence was limited	Implementation may be constrained by infrastructure requirements, costs, local ICU design and workflow compatibility. The added value of combining environmental modifications with other strategies remains uncertain
Quiet‐hour and workflow interventions	Scheduled quiet periods; clustering of care; minimising non‐urgent night‐time activities; reducing interruptions; light dimming	Aims to minimise avoidable night‐time disturbances through coordinated care and workflow adaptation	Implementation evidence reported improved adherence to selected noise‐reduction practices; direct effects on sleep and delirium were not established	Vulnerable to disruption by patient acuity and emergencies; requires flexible implementation, multidisciplinary coordination and staff adherence.
Staff engagement and culture change	Staff education; behavioural cues; leadership support; shared accountability; audit and feedback; acoustic dashboards	May support staff awareness, adherence and local adaptation through feedback and shared responsibility	Studies described noise‐related strain, staff experiences and implementation processes. Direct benefits for teamwork, well‐being and sustainability were insufficiently established	Frontline participation, leadership support and ongoing feedback may influence implementation feasibility. Their independent effects on adoption and sustainability require further evaluation
Multicomponent interventions	Combined alarm management, environmental modifications, quiet‐hour protocols, staff education and feedback systems	Aims to address interacting environmental, technological, behavioural and organisational contributors to ICU noise	Selected studies reported variable acoustic or practice‐related improvements. Comparative superiority and long‐term durability remain uncertain.	Local adaptation, interdisciplinary collaboration and continuous monitoring may support feasibility, although their independent contributions to intervention effectiveness and sustainability require further evaluation

*Note:* Proposed mechanisms and implementation considerations represent interpretations of the reviewed evidence and should not be regarded as established causal effects. Directly reported findings are distinguished from broader implications derived through the integrative synthesis.

### 3.2. Methodological Characteristics and Limitations of Included Publications

Methodological characteristics varied across the included publications. Primary observational and implementation studies provided information on acoustic exposure, alarm‐related outcomes, staff experiences and implementation processes, although several were conducted at single centres, lacked independent comparators or had limited follow‐up. Qualitative and participatory design studies contributed insights into nursing experiences and environmental design but were not suitable for estimating intervention effects. Review‐level sources provided broader contextual evidence, while their heterogeneity and potential overlap with primary studies limited independent confirmation of effectiveness.

Common limitations included small samples, variable intervention intensity, inconsistent outcome definitions, limited fidelity reporting and difficulty isolating the effects of individual components within bundled interventions. Review‐level sources strengthened contextual interpretation but were heterogeneous in scope, methods and transparency of reporting. They were therefore used to support the interpretation of field‐level patterns rather than to estimate independent intervention effects.

Because of heterogeneity in study design, intervention content, comparator use, outcome measurement and reporting, statistical pooling was not appropriate. Findings were interpreted narratively, with greater interpretive weight given to primary empirical and implementation studies when describing intervention effects. Qualitative and review‐level evidence were used to support the interpretation of nursing workflow, staff experience, alarm management practices, implementation barriers and the durability of change.

### 3.3. Overview of Reported Outcomes

Across the included studies, ICU noise and alarm burden were reported as both patient‐care and staff/workforce concerns. Patient‐related outcomes were most commonly linked to sleep disruption, reduced rest, sensory stress, delirium‐related concerns and environmental comfort. Staff‐related outcomes were most commonly linked to subjective noise exposure, noise‐related strain, cognitive workload, concentration difficulties, alarm fatigue, workflow interruption, and practical challenges in alarm response.

Findings varied across the included publications according to study design, intervention characteristics, outcome measures and implementation context. Primary empirical studies examined acoustic exposure, staff perceptions, alarm fatigue, observational patient‐outcome associations and selected implementation outcomes [7, 8, 10, 13, 16, 17]. Review‐level publications described a broader range of noise‐reduction and alarm‐management strategies, although their findings were not treated as independent confirmation of evidence represented by overlapping primary studies [3, 6, 11]. Selected interventions were associated with favourable acoustic or practice‐related outcomes, but methodological heterogeneity limited meaningful comparisons between strategies. In particular, the available evidence did not establish that multicomponent interventions were superior to single‐component approaches or that acoustic improvements consistently translated into measurable workforce or patient‐safety benefits.

Several publications highlighted challenges in maintaining noise‐management interventions over time. Workflow compatibility, staff engagement, leadership support, alarm governance and ongoing feedback emerged as potentially relevant implementation considerations, although their independent contributions to sustainability were not established.

### 3.4. Alarm Management Strategies

Alarm management strategies were a dominant focus in four studies. These included studies examining intelligent alarm management interventions, ICU nurses’ experiences with alarm management, alarm fatigue and alarm management responses and strategies. Interventions included individualised alarm thresholds, alarm parameter review, escalation protocols, remote or intelligent alarm surveillance, filtering of non‐actionable alarms and measures to improve alarm reliability.

Review‐level evidence described potential benefits of intelligent alarm‐management interventions, including reductions in false or non‐actionable alarms, shorter alarm duration, and improved responses to clinically important alerts [3]. However, these findings were derived from previously synthesised intervention studies and were not treated as independent evidence of effectiveness within the present review. Primary qualitative and mixed‐methods investigations described nurses′ alarm‐management experiences and strategies, including signal discrimination, team monitoring, reflective learning and behavioural or organisational coping approaches [8, 10]. Direct evidence that these strategies improve clinical surveillance, cognitive workload or patient safety remained limited.

Alarm management was closely linked to nursing workflow because nurses were frequently the first responders to bedside alarms. Poorly configured or poorly prioritised alarms contributed to workflow interruption, cognitive overload, alarm desensitisation and difficulty distinguishing clinically important alarms from low‐value notifications. Alarm‐management interventions, therefore, had both patient‐safety and workforce relevance.

Across studies, alarm‐management interventions appeared more feasible when supported by staff training, standardised protocols, interdisciplinary agreement, device interoperability, clear escalation pathways and ongoing governance.

### 3.5. Environmental Modifications

Environmental modifications were the dominant or explicit focus in four studies. These included studies on ICU soundscape modification, acoustic mapping, patient‐centred ICU design and built‐environment design. Strategies included sound‐absorbing materials, acoustic panels, flooring changes, door management, signage, spatial design, bed space redesign and broader healing‐environment approaches.

The contributing publications primarily characterised ICU acoustic exposure, environmental noise sources and considerations for recovery‐supportive ICU design [1, 2, 7, 12]. Acoustic monitoring identified routine bedside care activities and clinical equipment as contributors to environmental sound, while observational evidence examined associations between sensory exposure and delirium incidence. Participatory design and review‐level publications highlighted the potential relevance of environmental features, including acoustics, privacy, lighting and spatial configuration. However, these sources did not establish consistent causal effects of environmental modifications on sound exposure, patient comfort, sleep or clinical outcomes.

Environmental modifications may form part of a broader noise‐management approach, although their independent effectiveness and contribution within multicomponent interventions remain uncertain. Their feasibility and potential benefits should be evaluated in relation to local ICU design, clinical workflows and other concurrent noise‐management strategies.

### 3.6. Quiet‐Hour and Workflow Interventions

Quiet‐hour and workflow interventions were directly represented in one best‐practice implementation project and contributed to broader multicomponent or sound‐mapping evidence across four sources. These interventions included scheduled quiet periods, care clustering, minimising non‐urgent night‐time activities, reducing interruptions, dimming lights, promoting patient rest and reorganising bedside workflow to reduce unnecessary sound peaks.

Implementation evidence described improvements in adherence to selected noise‐reduction practices following structured quiet‐hour and workflow interventions [13]. However, achieving recommended environmental sound thresholds remained challenging. Although these strategies aim to support uninterrupted rest and reduce avoidable disturbances, the included implementation evidence did not establish consistent improvements in sleep quality, sleep fragmentation or delirium incidence. Future evaluations should therefore assess both implementation adherence and directly measured patient‐centred outcomes.

Quiet‐hour and workflow interventions were relevant to nursing practice because they involved care clustering, rest promotion, communication management, environmental control and prioritisation of non‐urgent tasks. Implementation was constrained by patient acuity, emergencies, staffing variability, competing clinical demands and the unpredictability of ICU workflow.

These interventions appeared most feasible when implemented flexibly, supported by multidisciplinary coordination, and adapted to real‐time clinical conditions rather than applied as rigid rules.

### 3.7. Staff Engagement and Culture Change

Staff engagement and culture change were represented across six contributing studies, including unit‐based noise management, nurses’ experiences with alarm management, quiet‐hour implementation, alarm fatigue, sustainable noise management programmes and qualitative syntheses of alarm management strategies. Strategies included staff education, behavioural cues, shared accountability, audit and feedback, acoustic dashboards, staff participation, leadership support and reinforcement of noise‐conscious behaviours.

Staff‐focused studies documented high perceived noise exposure, noise‐related strain, alarm fatigue and practical challenges associated with alarm management [8, 10, 16]. Implementation‐oriented publications also described staff education, participation, behavioural reinforcement and audit and feedback as components of noise‐management programmes [13, 17]. However, direct evidence demonstrating improvements in teamwork, occupational well‐being or long‐term behavioural change was limited.

Staff engagement and organisational support therefore emerged primarily as implementation considerations rather than established determinants of intervention effectiveness. For nursing management, the findings suggest that frontline participation, feedback and workflow compatibility warrant consideration when designing and evaluating Quiet ICU interventions. Their independent contributions to intervention adoption and sustainability require further investigation.

Staff education, ongoing feedback, leadership engagement and integration into routine unit processes were identified as potentially relevant implementation considerations. However, their individual contributions to intervention effectiveness and long‐term sustainability could not be established from the available evidence.

### 3.8. Multicomponent Bundled Approaches

Multicomponent bundled approaches were represented across four contributing sources, including structured quiet‐hour implementation, patient‐centred ICU design, systematic review evidence on noise‐mitigating interventions and a sustainable ICU noise‐management programme. These approaches combined alarm management, environmental modification, quiet‐hour protocols, staff education, behavioural expectations, workflow redesign, feedback systems and implementation monitoring.

Multicomponent approaches combined environmental, behavioural, workflow and organisational strategies to address several potential sources of ICU noise simultaneously. Selected implementation studies reported favourable changes in acoustic or practice‐related outcomes, although the magnitude and durability of these improvements varied [13, 17]. These approaches may offer practical advantages by addressing multiple interacting contributors to the ICU acoustic environment. However, the available evidence does not establish their superiority over single‐component interventions. Differences in intervention composition, study design, outcome measurement and follow‐up limited direct comparisons, and the independent contributions of individual bundle components could not be established. Further controlled and longitudinal evaluations are therefore needed to determine their comparative effectiveness, feasibility and sustainability.

Bundled approaches also created interpretive challenges. The evidence did not allow firm conclusions about which individual components were most effective because intervention packages varied across studies and were often evaluated as whole programmes. Follow‐up duration varied, and the durability of benefit was not consistently demonstrated. One implementation study reported modest post‐intervention reductions in sound levels, but gains were less durable at follow‐up and smaller after workload adjustment.

These approaches addressed multiple domains relevant to nursing management, including alarm relevance, staff behaviour, workflow design, unit leadership and maintenance of practice change. However, their comparative advantages over individual interventions were not established.

### 3.9. Cross‐Cutting Nursing Management Findings

Across the thematic categories, several domains relevant to nursing management were identified, including alarm burden, staff experiences, care coordination, workflow feasibility and implementation processes. However, these domains were not consistently evaluated as direct outcomes across the included publications. Alarm‐management research primarily informed understanding of alarm‐related experiences and selected alarm performance measures, whereas quiet‐hour, environmental, and participatory design publications contributed information concerning acoustic conditions, care practices and implementation considerations.

The synthesis suggests that workflow compatibility, frontline participation, escalation procedures and organisational support may influence the feasibility of Quiet ICU interventions. These relationships were derived from patterns across heterogeneous publications rather than established through direct comparative evaluations of nursing management strategies. Consequently, the contribution of specific leadership and governance mechanisms to intervention effectiveness and long‐term sustainability remains uncertain.

### 3.10. Implementation Barriers and Maintenance of Change

Implementation barriers were reported across all intervention categories. Common barriers included patient acuity, emergencies, competing clinical priorities, staffing variability, inconsistent adherence, limited follow‐up, variable unit support and difficulty maintaining changes over time. Technical interventions were limited when poorly integrated into bedside practice, while behavioural interventions were limited when dependent mainly on individual motivation.

The distinction between short‐term improvement and sustained implementation emerged as an important consideration. Although selected studies reported favourable acoustic, practice‐related or staff‐perception outcomes, evidence concerning long‐term maintenance remained limited [13, 16, 17]. Audit and feedback, staff participation, local adaptation, leadership support and ongoing monitoring were identified as potential implementation considerations. However, the independent contributions of these components to intervention sustainability were not established.

Overall, the evidence identified several potentially useful approaches for addressing ICU noise and alarm burden, including alarm management, environmental modification, workflow adaptation, staff engagement and multicomponent implementation. Their comparative effectiveness and sustainability remain uncertain. From a nursing management perspective, future evaluation should determine whether these approaches improve acoustic conditions while preserving alarm recognition, clinical surveillance and compatibility with routine ICU workflows.

## 4. Discussion

### 4.1. Principal Findings

This integrative review indicates that ICU noise and alarm burden should be understood as nursing management, patient‐safety and workforce issues rather than as narrow environmental problems [20, 21]. Across the included evidence, excessive sound exposure was shaped by the interaction of alarm systems, bedside workflow, staff communication, patient acuity, environmental design and organisational practices [1, 3, 4, 12]. These findings highlight the potential value of addressing multiple sources of ICU noise and alarm burden within a coordinated implementation framework. However, the available evidence does not establish that multicomponent interventions are more effective or sustainable than single‐component approaches [6, 13, 17].

A key contribution of this review is the reframing of Quiet ICU interventions within a broader nursing management perspective. Sustainable ICU noise reduction may require attention to more than acoustic outcomes, including alarm governance, workflow integration, staff engagement, patient surveillance, escalation procedures and managerial accountability. However, the independent contributions of these components to intervention effectiveness and long‐term sustainability remain uncertain.

Because this review integrated primary empirical studies, implementation studies, qualitative evidence and review‐level sources, the findings should be interpreted as an integrative synthesis of patterns across a heterogeneous field rather than as pooled estimates of intervention effectiveness.

### 4.2. Systems‐Based Interpretation

The findings can be interpreted through established safety and management frameworks. From a structure‐process‐outcome perspective, ICU design, alarm configuration, staffing support, technology infrastructure and local policy represent structural conditions of care; alarm review, care clustering, quiet‐hour implementation, communication practices and behavioural reinforcement represent care processes; and patient sleep, delirium‐related outcomes, staff fatigue, cognitive load, alarm fatigue and perceived safety represent relevant outcomes [20]. This framework highlights that ICU noise reduction is unlikely to be maintained if interventions focus only on physical sound control while neglecting work processes and staff experience.

The Swiss Cheese Model provides a complementary safety perspective by showing how risk may persist when multiple weak layers coexist, including poor alarm configuration, frequent overnight interruptions, unclear ownership, inconsistent staff engagement and limited implementation support [21]. In this context, addressing environmental, technological, behavioural and organisational factors within a coordinated framework may support the feasibility of Quiet ICU strategies, although evidence establishing the superiority of integrated approaches over isolated interventions remains limited [3, 12, 13]. As shown in Figure 2, a systems‐based Quiet ICU framework positions noise reduction as a coordinated nursing management strategy linking structural conditions, bedside processes, staff behaviours, outcomes and continuous feedback.

**FIGURE 2:**
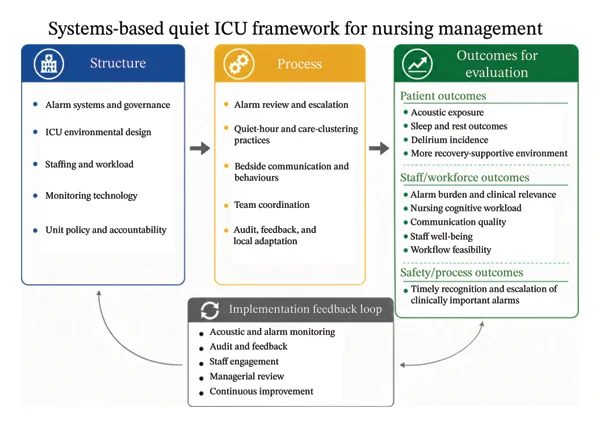
Systems‐based Quiet ICU framework for nursing management. The framework conceptualises ICU noise and alarm burden as outcomes of interacting structural conditions, bedside processes, staff behaviours and organisational factors. It identifies acoustic exposure, alarm burden, nursing workload, clinical surveillance, patient‐related outcomes and implementation sustainability as potential domains for evaluation. The proposed relationships are intended to guide future research and quality‐improvement activities and should not be interpreted as established causal effects demonstrated by the included evidence.

### 4.3. Alarm Burden, Nursing Surveillance and Recognition of Clinical Deterioration

An important consideration arising from this review is the potential relationship between alarm burden and nursing surveillance. Nurses integrate physiological monitoring, clinical assessments and contextual patient information to recognise changes in patient condition and prioritise clinical responses. Frequent non‐actionable alarms may increase competing demands for attention and complicate the discrimination of clinically important signals. Nevertheless, reductions in audible alarm frequency or environmental sound should not automatically be interpreted as improvements in surveillance performance or patient safety.

Evidence from broader inpatient nursing research highlights the potential importance of nursing assessment information in recognising patients at risk of clinical deterioration. Cesare and Cocchieri [22] examined associations between nursing care complexity and intra‐hospital or ICU transfers among hospitalised children. Subsequently, Cesare et al. [23] investigated the predictive relevance of nursing diagnostic profiles for ICU transfer risk among adult and paediatric hospitalised patients using machine‐learning methods. Although these studies did not evaluate Quiet ICU interventions, their findings provide relevant contextual evidence concerning the role of nursing‐generated clinical information in deterioration‐risk assessment.

These findings identify an important direction for future research. Quiet ICU interventions should be evaluated not only for their effects on environmental sound and alarm frequency but also for their potential consequences for nursing situational awareness, alarm recognition, response latency and escalation reliability. Patient‐safety balancing measures, including missed or delayed clinically important alerts, should be incorporated to determine whether reductions in alarm burden can be achieved without compromising clinical surveillance or timely recognition of deterioration.

### 4.4. Sustainability and Implementation

A central finding was the gap between short‐term improvement and maintained practice change. Several interventions showed early promise, but durability was less consistently demonstrated [13, 17]. This should not be interpreted simply as poor staff motivation or limited awareness. Instead, the evidence suggests that interventions were less durable when poorly aligned with bedside workflow, required repeated manual effort, lacked clear operational ownership or were not supported by feedback and managerial reinforcement [9, 10, 16, 17].

For nurse leaders, this is a practical management issue. Noise‐reduction strategies are unlikely to be sustained if they add to the workload of already pressured bedside care, conflict with urgent clinical priorities or fail to address alarm burden, cognitive strain and workflow disruption. The central management challenge is not whether quieter practice is desirable, but whether it can be embedded into routine ICU work without compromising surveillance, escalation or staff workload.

### 4.5. Nurses as Affected Stakeholders and Implementation Agents

This review highlights the dual role of nurses as both affected stakeholders and implementation agents. Nurses experience sustained exposure to ICU noise and alarm burden while also carrying responsibility for patient surveillance, alarm response, communication, care coordination and rest promotion. Quiet ICU interventions, therefore, cannot be designed solely as environmental or technical solutions. They must be operationally feasible for bedside nurses and supported by managers who can align staffing realities, escalation processes, alarm‐management expectations and audit‐feedback mechanisms.

Involving nurses in intervention design, local adaptation and evaluation may enhance the acceptability and feasibility of Quiet ICU strategies. However, direct comparative evidence establishing the effectiveness of participatory approaches over top‐down implementation remains limited. Such involvement may be particularly relevant in high‐acuity settings, where patient deterioration, urgent procedures, staffing variation and competing priorities can rapidly disrupt quiet‐hour protocols or behavioural noise‐reduction strategies. For nursing management, the practical challenge is to design interventions that preserve safety, maintain alarm recognition, support patient surveillance and reduce avoidable workload, rather than simply asking staff to ‘be quieter’.

### 4.6. Implications for Nursing Management

The nursing management implications identified through this integrative review should be distinguished from outcomes directly evaluated in the included publications. Most empirical investigations examined acoustic exposure, alarm‐related outcomes, staff experiences or selected implementation processes. Relatively few studies directly assessed nursing leadership effectiveness, managerial accountability, workforce well‐being, clinical surveillance or sustained organisational change. Accordingly, the following considerations represent implications derived from the integrative synthesis rather than demonstrated effects of specific management interventions. Nurse managers may consider incorporating noise and alarm management into existing patient‐safety and quality‐improvement structures through defined alarm responsibilities, frontline nursing involvement, workflow integration and evaluation of acoustic, workforce and clinical safety outcomes.

Second, nurses should be actively involved in intervention design and local adaptation so that strategies reflect bedside workflow, patient acuity, staffing patterns and real‐time clinical priorities [4, 9]. Third, alarm‐management policies should be practical, individualised and supported by education, audit, feedback, interdisciplinary agreement and managerial reinforcement [3, 10]. Fourth, quiet‐hour protocols should remain flexible enough to accommodate clinical deterioration and urgent care while protecting patient rest whenever feasible [6, 13].

Finally, evaluation should extend beyond decibel reduction. Nursing management outcomes should include alarm burden, cognitive load, concentration, communication, teamwork, workflow feasibility, staff well‐being, perceived safety and maintenance of practice change [8, 16, 17]. These outcomes capture whether Quiet ICU interventions are not only acoustically effective but also feasible and safe within everyday ICU nursing practice.

### 4.7. Future Research

Future research should move beyond short‐term before‐and‐after evaluations and adopt more robust, multisite and longitudinal designs. Studies should report intervention fidelity, implementation context, staff engagement, visible nursing leadership, leadership accountability and maintenance of change over time. Because many interventions are bundled, future studies should describe intervention components clearly and, where possible, examine which components contribute most to observed improvements.

Greater consistency in outcome measurement is also needed. Decibel reduction remains important but should be complemented by patient‐centred, workforce and system‐level outcomes, including alarm fatigue, cognitive workload, communication quality, workflow disruption, staff well‐being, perceived feasibility and sustainability. This broader approach would better support decisions about implementation, adaptation and scale‐up in ICU nursing management.

### 4.8. Strengths and Limitations

This review synthesised recent evidence across alarm management, environmental modification, workflow redesign, staff engagement and implementation culture while maintaining explicit attention to critical care nursing management. The integrative design enabled the inclusion of practice‐oriented evidence that may not have been captured within narrower review frameworks and allowed intervention effects to be considered alongside feasibility, leadership requirements, workforce implications and sustainability.

Several limitations warrant consideration. First, the search was restricted to three electronic databases, English‐language peer‐reviewed publications and a defined publication period, potentially excluding relevant evidence from other sources and grey literature. Second, the search focused primarily on ICU acoustic exposure, noise reduction and alarm management rather than comprehensively examining the broader literature concerning nursing leadership, workforce well‐being and organisational sustainability. Furthermore, the database search strategies required intervention‐ or management‐related terminology, which may have limited the retrieval of observational acoustic studies, descriptive investigations, and other contextual evidence eligible under the broader integrative‐review criteria. Third, the integrative review methodology differed from the original PROSPERO‐registered systematic‐review protocol in its eligibility criteria, evidence sources, search scope and appraisal methods. Fourth, methodological heterogeneity, differences in outcome measurement, limited comparator data and inconsistent follow‐up constrained comparisons between intervention strategies [3, 6, 12, 13]. The inclusion of observational, qualitative, design‐oriented, implementation and review‐level publications provided a broader understanding of ICU noise and alarm burden but limited the extent to which comparative intervention‐effect conclusions could be drawn. Although overlapping evidence was explicitly considered, complete overlap ascertainment was not possible for all review‐level publications. Furthermore, Armbruster et al. [16] and Witek et al. [17] reported complementary outcomes from the same registered ICU noise‐management programme and therefore did not represent independent intervention evaluations. As the programme included both adult and neonatal ICUs, findings were interpreted cautiously regarding their generalisability to adult critical care. Where adult‐specific outcomes could not be distinguished from aggregated programme findings, the results were not assumed to represent adult ICU intervention effects exclusively.

For these reasons, conclusions about intervention effectiveness and long‐term maintenance should be interpreted as integrative and directional rather than definitive. The value of this review lies in clarifying patterns across a complex evidence base and identifying implications for nursing leadership, unit management, workforce well‐being and sustainable critical care practice.

## 5. Conclusion

This integrative review identified alarm‐management strategies, environmental modifications and quiet‐hour or workflow interventions as approaches relevant to addressing noise and alarm burden in adult ICUs. Staff engagement and multicomponent implementation emerged as additional considerations for nursing management. However, the available evidence was heterogeneous and insufficient to establish the comparative superiority or long‐term sustainability of particular intervention strategies. Direct evidence linking improvements in the ICU acoustic environment with nursing workload, workforce well‐being, clinical surveillance and patient‐safety outcomes remained limited.

For nursing management, the findings highlight the importance of considering ICU noise and alarm burden within broader patient‐safety, workflow and workforce‐improvement initiatives. Nurse leaders may consider context‐sensitive interventions supported by clearly defined alarm responsibilities, frontline staff involvement and ongoing evaluation of clinical, acoustic and implementation outcomes. Further research is needed to establish whether improvements in ICU acoustic conditions translate into meaningful and sustained benefits for nursing surveillance, workforce experience and patient care.

## Author Contributions

Author contributions are reported using the Contributor Roles Taxonomy (CRediT). Wei Jun Dan Ong: conceptualization, methodology, investigation, data curation, formal analysis, visualization, writing–original draft and project administration. Michael Camba Vidanes: investigation, data curation, validation, formal analysis and writing–review and editing. Woon Hean Keenan Chong: validation, supervision and writing–review and editing. Faheem Ahmed Khan: methodology, supervision, validation and writing–review and editing. Patricia Leong: resources and writing–review and editing. Lawrence Ace Matunan Azul: investigation, resources, project administration and writing–review and editing. Adrian Ujin Yap: methodology, supervision, validation and writing–review and editing.

## Funding

This research received no specific grant from any funding agency in the public, commercial or not‐for‐profit sectors.

## Disclosure

All authors reviewed and approved the final manuscript.

## Ethics Statement

This integrative review synthesised evidence from previously published literature and did not involve primary participant recruitment, collection of identifiable patient information or delivery of a clinical intervention. Institutional ethics approval and informed consent were therefore not required, and an ethics approval number was not applicable. A related systematic‐review protocol was registered with PROSPERO on 23 October 2025 (CRD420251172154). The methodological differences between the registered protocol and the completed integrative review are described in the Methods section.

## Conflicts of Interest

The authors declare no conflicts of interest.

## Supporting Information

Additional supporting information can be found online in the Supporting Information section.

## Supporting information


**Supporting Information 1** Supporting Table S1 provides detailed search strategies used in PubMed, Scopus and CINAHL, including search terms and eligibility restrictions. Supporting Table S2 presents the overlap assessment between primary studies and review‐level publications to identify potential duplication of evidence. Supporting Table S3 summarises the methodological appraisal of the 13 included publications, including appraisal approaches, key limitations and contributions to the integrative synthesis.

**Supporting Information 2** PRISMA_2020_checklist_completed_Quiet_ICU (3).

## Data Availability

Data sharing is not applicable to this article because no new primary datasets were generated or analysed. The evidence synthesised in this integrative review was derived from previously published studies cited in the manuscript. The review extraction matrix and appraisal summaries are available from the corresponding author upon reasonable request.
