# Supplementary material for: Reducing Noise and Alarm Burden in Adult Intensive Care Units: An Integrative Review of Interventions and Implications for Nursing Management
Source: J Nurs Manag. 2026 Oct 29;2026:9617407. doi: 10.1155/jonm/9617407 (PMC13717587; doi:10.1155/jonm/9617407)
Supplement: Supplementary file 1 [file JONM-2026-9617407-s001.docx]

**Table S1:** Detailed search strategy of individual databases

| **Database** | **Search strategy** |
| --- | --- |
| PubMed | (("intensive care unit"[Title/Abstract] OR "critical care"[Title/Abstract] OR ICU[Title/Abstract]) AND ("noise"[Title/Abstract] OR "sound level"[Title/Abstract] OR "sound pressure"[Title/Abstract] OR acoustics[Title/Abstract] OR "acoustic environment"[Title/Abstract] OR "alarm fatigue"[Title/Abstract] OR "alarm burden"[Title/Abstract] OR "clinical alarm"[Title/Abstract] OR "monitor alarm"[Title/Abstract]) AND ("intervention"[Title/Abstract] OR "alarm management"[Title/Abstract] OR "noise reduction"[Title/Abstract] OR "noise control"[Title/Abstract] OR "quiet hour"[Title/Abstract] OR "quiet hours"[Title/Abstract] OR "quiet time"[Title/Abstract] OR "sleep promotion"[Title/Abstract] OR "sound masking"[Title/Abstract] OR "environmental modification"[Title/Abstract] OR workflow[Title/Abstract] OR implementation[Title/Abstract])) AND english[Language] AND ("2019/01/01"[Date - Publication] : "2025/08/31"[Date - Publication]) |
| Scopus | TITLE-ABS-KEY("intensive care unit" OR "critical care" OR ICU) AND TITLE-ABS-KEY(noise OR "sound level" OR "sound pressure" OR acoustics OR "acoustic environment" OR "alarm fatigue" OR "alarm burden" OR "clinical alarm" OR "monitor alarm") AND TITLE-ABS-KEY(intervention OR "alarm management" OR "noise reduction" OR "noise control" OR "quiet hour" OR "quiet hours" OR "quiet time" OR "sleep promotion" OR "sound masking" OR "environmental modification" OR workflow OR implementation) AND PUBYEAR > 2018 AND PUBYEAR < 2026 AND LIMIT-TO(LANGUAGE, "English") AND (LIMIT-TO(DOCTYPE, "ar") OR LIMIT-TO(DOCTYPE, "re")) |
| CINAHL | ((TI "intensive care unit" OR AB "intensive care unit" OR TI "critical care" OR AB "critical care" OR TI ICU OR AB ICU) AND (TI noise OR AB noise OR TI "sound level" OR AB "sound level" OR TI "sound pressure" OR AB "sound pressure" OR TI acoustics OR AB acoustics OR TI "acoustic environment" OR AB "acoustic environment" OR TI "alarm fatigue" OR AB "alarm fatigue" OR TI "alarm burden" OR AB "alarm burden" OR TI "clinical alarm" OR AB "clinical alarm" OR TI "monitor alarm" OR AB "monitor alarm") AND (TI intervention OR AB intervention OR TI "alarm management" OR AB "alarm management" OR TI "noise reduction" OR AB "noise reduction" OR TI "noise control" OR AB "noise control" OR TI "quiet hour" OR AB "quiet hour" OR TI "quiet hours" OR AB "quiet hours" OR TI "quiet time" OR AB "quiet time" OR TI "sleep promotion" OR AB "sleep promotion" OR TI "sound masking" OR AB "sound masking" OR TI "environmental modification" OR AB "environmental modification" OR TI workflow OR AB workflow OR TI implementation OR AB implementation)) Limiters: English Language; Published Date: 20190101-20250831; Peer Reviewed |

**Notes.** The search used a publication-year restriction of 2019-2025. The eligibility period for the integrative review ended on 31 August 2025. Records published after this date were excluded during eligibility assessment to maintain consistency across the databases. Backward citation screening of all included articles was performed manually. Reference lists were reviewed to identify additional potentially eligible studies that were not retrieved through the primary database search. Any newly identified studies underwent the same eligibility assessment process as database-retrieved records.

**Eligibility framing used during searching and screening**

Studies were considered potentially relevant if they:

- focused on adult ICU or critical care settings
- addressed noise reduction, alarm burden, or related implementation strategies
- reported patient-, staff-, or system-level outcomes
- were published in peer-reviewed journals
- were written in English

Studies were excluded if they:

- focused exclusively on neonatal or paediatric ICUs
- were conducted outside ICU settings
- did not address an intervention, implementation strategy, or contextual factor related to ICU noise or alarm burden

**Supplemental Table S2. Overlap between primary reports and review-level sources**

Published-source comparison of five reviews with eight primary reports.

| **Review-level source** | **Armbruster (2023)** | **Lu (2024)** | **Sangari (2021)** | **Souza (2022)** | **Tahvili (2025)** | **Tronstad (2023)** | **Uçak (2025)** | **Witek (2025)** |
| --- | --- | --- | --- | --- | --- | --- | --- | --- |
| Li et al. (2023) | T | T | N | N | T | T | T | T |
| Pal et al. (2022) | T | T | U | U | T | T | T | T |
| Verderber et al. (2021) | T | T | T | T | T | T | T | T |
| Vreman et al. (2023) | T | T | N | Y | T | T | T | T |
| Xu et al. (2025) | N | T | N | N | T | N | T | T |

**Notes.** Y = confirmed inclusion as a study within the review; N = not identified in the available included-study list; T = published after the review’s reported search window; U = overlap could not be established from the published information. An N does not mean the paper was never cited for background.

**Interpretation.** One confirmed study-within-review overlap was identified: Souza et al. (2022) was included in Vreman et al. (2023). Armbruster et al. (2023) and Witek et al. (2025) are separate outcome reports from the same registered German ICU noise-management programme (DRKS00025835). Primary-study findings were not counted again as independent effects when described by a review; staff and acoustic outcomes from the shared programme were interpreted as complementary rather than independent replications. Where a review did not provide a reproducible included-study inventory, overlap remained indeterminate and the review was used for contextual interpretation only.

**Supplemental Table S3.** *Methodological appraisal of included studies*

| Publication | Study design / evidence type | Appraisal instrument / approach | Key methodological findings / limitations | Contribution to integrative synthesis |
| --- | --- | --- | --- | --- |
| Armbruster et al. (2023) | Primary; prospective longitudinal staff survey | JBI Checklist for Cohort Studies | Single-centre observational staff reports; possible temporal confounding and limited separation of exposure from programme effects. Includes mixed adult and neonatal units. | Staff-reported noise experience; complementary outcome report from the Witek programme. |
| Li et al. (2023) | Review-level; integrative review | JBI Checklist for Systematic Reviews and Research Syntheses | Different alarm systems and underlying intervention designs limit direct cross-study comparisons and certainty of effectiveness. | Secondary context for alarm-management approaches; not independent intervention-effect evidence. |
| Lu et al. (2024) | Primary; qualitative interview study | JBI Checklist for Qualitative Research | Small, context-specific nursing sample; findings concern experiences and strategies rather than measured intervention effects. | Insights into nurses’ alarm-management experience and workflow. |
| Pal et al. (2022) | Review-level; narrative review | Structured narrative-review methodological appraisal | Search, selection and included-study inventory are not sufficiently reproducible for a complete overlap audit. | General conceptual background; not independent corroboration of effects. |
| Sangari et al. (2021) | Primary; observational sound/light–delirium association | JBI Checklist for Cohort Studies | No acoustic intervention assigned; associations may reflect residual confounding and do not establish causation. | Contextual patient-outcome association, not noise-intervention effectiveness. |
| Souza et al. (2022) | Primary; best-practice implementation project | JBI Checklist for Quasi-Experimental Studies | Local practice-audit improvement without a strong independent comparator; susceptibility to implementation and temporal confounding. | Direct implementation findings; also represented in the Vreman review. |
| Tahvili et al. (2025) | Primary; descriptive acoustic monitoring | Structured descriptive observational and acoustic-methods appraisal | Limited setting and observation period; characterised sound sources but did not evaluate an intervention. | Contextual ICU acoustic-exposure evidence, not an intervention-effect estimate. |
| Tronstad et al. (2023) | Primary; multistage participatory ICU design study | Mixed Methods Appraisal Tool (MMAT), 2018, mixed-methods category | Design-centred development and early environmental evaluation; no robust isolation of clinical effects from redesign components. | Implementation and participatory-design insights; not a literature review. |
| Uçak et al. (2025) | Primary; convergent mixed-methods study | Mixed Methods Appraisal Tool (MMAT), 2018, mixed-methods category | Site-specific nursing sample; integration and transferability of qualitative and quantitative strands constrain interpretation. | Describes nurses’ alarm fatigue and coping responses; not a causal intervention trial. |
| Verderber et al. (2021) | Review-level; broad built-environment literature review | Structured narrative/integrative literature-review methodological appraisal | Heterogeneous architectural and clinical sources; not designed to estimate one ICU noise-intervention effect. | Conceptual background for the healing ICU environment. |
| Vreman et al. (2023) | Review-level; systematic review | JBI Checklist for Systematic Reviews | Heterogeneity and limitations of underlying interventions constrain comparison; includes Souza et al. (2022). | Secondary evidence synthesis; overlapping primary findings not double-counted. |
| Witek et al. (2025) | Primary; non-randomised before–after programme evaluation | JBI Checklist for Quasi-Experimental Studies | Context-dependent sound changes, workload-related confounding, limited durability; mixed adult and neonatal units. | Direct acoustic programme evaluation; shares registered programme with Armbruster. |
| Xu et al. (2025) | Review-level; qualitative systematic review/meta-synthesis | JBI Checklist for Systematic Reviews | Findings represent synthesised perceptions across heterogeneous qualitative settings, not directly observed alarm-intervention effects. | Secondary contextual themes concerning nurses’ alarm experiences. |

**Notes.** Two reviewers independently assessed the methodological quality of included publications using design-appropriate Joanna Briggs Institute (JBI) critical appraisal instruments, the Mixed Methods Appraisal Tool (MMAT), or structured methodological appraisal criteria, as applicable. Disagreements were resolved through discussion or consultation with a third reviewer. The table presents the principal methodological limitations identified during appraisal. These findings informed the interpretation of the integrative synthesis but were not converted into numerical quality scores or formal certainty-of-evidence ratings. Review-level publications were used primarily for contextual interpretation. Souza et al. (2022) was also included in Vreman et al. (2023), while Armbruster et al. (2023) and Witek et al. (2025) reported complementary outcomes from the same registered ICU noise-management programme.
