# Supplementary material for: Reducing Noise and Alarm Burden in Adult Intensive Care Units: An Integrative Review of Interventions and Implications for Nursing Management
Source: J Nurs Manag. 2026 Oct 29;2026:9617407. doi: 10.1155/jonm/9617407 (PMC13717587; doi:10.1155/jonm/9617407)
Supplement: Supplementary file 2 [file JONM-2026-9617407-s002.docx]

| **Section and Topic** | **Item #** | **Checklist item** | **Location where item is reported** |
| --- | --- | --- | --- |
| **TITLE** | | |  |
| Title | 1 | Identify the report as a systematic review. | Title identifies an integrative review; PRISMA 2020 principles adapted where applicable |
| **ABSTRACT** | | |  |
| Abstract | 2 | See the PRISMA 2020 for Abstracts checklist. | Abstract: structured Aim, Background, Evaluation, Key issues, Conclusion and Implications for Nursing Management. |
| **INTRODUCTION** | | |  |
| Rationale | 3 | Describe the rationale for the review in the context of existing knowledge. | Introduction: environmental, patient-safety and nursing-management rationale and gaps in prior reviews. |
| Objectives | 4 | Provide an explicit statement of the objective(s) or question(s) the review addresses. | Introduction: aims and explicit review question. |
| **METHODS** | | |  |
| Eligibility criteria | 5 | Specify the inclusion and exclusion criteria for the review and how studies were grouped for the syntheses. | Methods: Study selection- eligibility criteria, evidence types and evidence roles; Methods-Data analysis, |
| Information sources | 6 | Specify all databases, registers, websites, organisations, reference lists and other sources searched or consulted to identify studies. Specify the date when each source was last searched or consulted. | Methods: Search strategy; Supplemental Table S1. PubMed, Scopus, CINAHL and backward reference screening. Final database search 31 Jan 2026; eligible publication period Jan 2019–31 Aug 2025. |
| Search strategy | 7 | Present the full search strategies for all databases, registers and websites, including any filters and limits used. | Methods: Search strategy and Supplemental Table S1: full PubMed, Scopus and CINAHL strings and limits. |
| Selection process | 8 | Specify the methods used to decide whether a study met the inclusion criteria of the review, including how many reviewers screened each record and each report retrieved, whether they worked independently, and if applicable, details of automation tools used in the process. | Methods: Study selection- Covidence, two independent screeners, full-text eligibility and arbitration by a third reviewer |
| Data collection process | 9 | Specify the methods used to collect data from reports, including how many reviewers collected data from each report, whether they worked independently, any processes for obtaining or confirming data from study investigators, and if applicable, details of automation tools used in the process. | Methods: Data extraction |
| Data items | 10a | List and define all outcomes for which data were sought. Specify whether all results that were compatible with each outcome domain in each study were sought (e.g. for all measures, time points, analyses), and if not, the methods used to decide which results to collect. | Methods: Data extraction- patient, staff, acoustic, alarm, system and implementation outcome domains. |
|  | 10b | List and define all other variables for which data were sought (e.g. participant and intervention characteristics, funding sources). Describe any assumptions made about any missing or unclear information. | Methods: Data extraction- authors, year, design, setting, sample, intervention, comparator, outcomes, barriers, appraisal. |
| Study risk of bias assessment | 11 | Specify the methods used to assess risk of bias in the included studies, including details of the tool(s) used, how many reviewers assessed each study and whether they worked independently, and if applicable, details of automation tools used in the process. | Methods: Data evaluation, JBI/MMAT and design-informed narrative approaches, two independent reviewers and resolution procedures. Study-level findings and named instruments: Supplemental Table S3. |
| Effect measures | 12 | Specify for each outcome the effect measure(s) (e.g. risk ratio, mean difference) used in the synthesis or presentation of results. | Methods: Statistical analysis. Individual study results reported narratively (Table 1). |
| Synthesis methods | 13a | Describe the processes used to decide which studies were eligible for each synthesis (e.g. tabulating the study intervention characteristics and comparing against the planned groups for each synthesis (item #5)). | Methods: Study selection and Data analysis |
|  | 13b | Describe any methods required to prepare the data for presentation or synthesis, such as handling of missing summary statistics, or data conversions. | Methods: Data extraction and Data analysis |
|  | 13c | Describe any methods used to tabulate or visually display results of individual studies and syntheses. | Main Table 1: characteristics and findings of all 13 publications. Main Table 2: thematic synthesis. Figure 1: selection; Figure 2: proposed conceptual framework; Supplemental Tables S2 & S3. |
|  | 13d | Describe any methods used to synthesize results and provide a rationale for the choice(s). If meta-analysis was performed, describe the model(s), method(s) to identify the presence and extent of statistical heterogeneity, and software package(s) used. | Methods: Statistical analysis, thematic narrative synthesis; no meta-analysis due to heterogeneous designs, interventions, and outcomes. |
|  | 13e | Describe any methods used to explore possible causes of heterogeneity among study results (e.g. subgroup analysis, meta-regression). | N/A: heterogeneity explored narratively; no subgroup/meta-regression. |
|  | 13f | Describe any sensitivity analyses conducted to assess robustness of the synthesized results. | N/A: no sensitivity analysis because no statistical pooling. |
| Reporting bias assessment | 14 | Describe any methods used to assess risk of bias due to missing results in a synthesis (arising from reporting biases). | N/A/not conducted: no formal reporting-bias assessment. |
| Certainty assessment | 15 | Describe any methods used to assess certainty (or confidence) in the body of evidence for an outcome. | N/A/not conducted: no GRADE/formal certainty assessment; methodological appraisal used instead. |
| **RESULTS** | | |  |
| Study selection | 16a | Describe the results of the search and selection process, from the number of records identified in the search to the number of studies included in the review, ideally using a flow diagram. | Results: Search results, and Figure 1 |
|  | 16b | Cite studies that might appear to meet the inclusion criteria, but which were excluded, and explain why they were excluded. | Results, Search results and Characteristics of Included Studies; Figure 1. Reasons for all 33 full-text exclusions are summarised. No excluded publications were identified that appeared to satisfy the final eligibility criteria and required individual explanation. |
| Study characteristics | 17 | Cite each included study and present its characteristics. | Main Table 1: all 13 included publications with design, setting, sample, focus, findings and limitations. |
| Risk of bias in studies | 18 | Present assessments of risk of bias for each included study. | Results: Methodological characteristics, Main Table 1, Supplemental Table S3 |
| Results of individual studies | 19 | For all outcomes, present, for each study: (a) summary statistics for each group (where appropriate) and (b) an effect estimate and its precision (e.g. confidence/credible interval), ideally using structured tables or plots. | Main Table 1, and Results thematic subsections, summarise findings. |
| Results of syntheses | 20a | For each synthesis, briefly summarise the characteristics and risk of bias among contributing studies. | Results: Methodological characteristics; Results, and Supplemental Table S3. Distinguishes primary findings from review-level context. |
|  | 20b | Present results of all statistical syntheses conducted. If meta-analysis was done, present for each the summary estimate and its precision (e.g. confidence/credible interval) and measures of statistical heterogeneity. If comparing groups, describe the direction of the effect. | N/A: no meta-analysis/statistical synthesis. |
|  | 20c | Present results of all investigations of possible causes of heterogeneity among study results. | N/A: no statistical heterogeneity investigation. |
|  | 20d | Present results of all sensitivity analyses conducted to assess the robustness of the synthesized results. | N/A: no sensitivity analysis. |
| Reporting biases | 21 | Present assessments of risk of bias due to missing results (arising from reporting biases) for each synthesis assessed. | N/A: no reporting-bias assessment. |
| Certainty of evidence | 22 | Present assessments of certainty (or confidence) in the body of evidence for each outcome assessed. | N/A: no formal certainty assessment; appraisal-informed interpretation in Results/Tables 1–2. |
| **DISCUSSION** | | |  |
| Discussion | 23a | Provide a general interpretation of the results in the context of other evidence. | Discussion: Principal findings; Systems-based interpretation; Sustainability and implementation; Nurses as stakeholders/agents. |
|  | 23b | Discuss any limitations of the evidence included in the review. | Discussion: Strengths and limitations |
|  | 23c | Discuss any limitations of the review processes used. | Discussion: Strengths and limitations |
|  | 23d | Discuss implications of the results for practice, policy, and future research. | Discussion: Implications for nursing management; Future research; Conclusion. |
| **OTHER INFORMATION** | | |  |
| Registration and protocol | 24a | Provide registration information for the review, including register name and registration number, or state that the review was not registered. | Methods—Design: Related systematic review protocol registered PROSPERO 23 Oct 2025, CRD420251172154. |
|  | 24b | Indicate where the review protocol can be accessed, or state that a protocol was not prepared. | A related systematic-review protocol was registered with PROSPERO; no separate integrative-review protocol is reported |
|  | 24c | Describe and explain any amendments to information provided at registration or in the protocol. | Differences between the registered systematic review and completed integrative review are disclosed. |
| Support | 25 | Describe sources of financial or non-financial support for the review, and the role of the funders or sponsors in the review. | Funding Statement |
| Competing interests | 26 | Declare any competing interests of review authors. | Conflict of Interest Statement |
| Availability of data, code and other materials | 27 | Report which of the following are publicly available and where they can be found: template data collection forms; data extracted from included studies; data used for all analyses; analytic code; any other materials used in the review. | Data Availability Statement |

Locations are given by manuscript section/table/figure rather than page number.

*From:*  Page MJ, McKenzie JE, Bossuyt PM, Boutron I, Hoffmann TC, Mulrow CD, et al. The PRISMA 2020 statement: an updated guideline for reporting systematic reviews. BMJ 2021;372:n71. doi: 10.1136/bmj.n71
